# Genetic diversity and signatures of selection in Icelandic horses and Exmoor ponies

**DOI:** 10.1186/s12864-024-10682-8

**Published:** 2024-08-08

**Authors:** Heiðrún Sigurðardóttir, Michela Ablondi, Thorvaldur Kristjansson, Gabriella Lindgren, Susanne Eriksson

**Affiliations:** 1 Department of Animal Biosciences, Swedish University of Agricultural Sciences, P.O. Box 7023, Uppsala, 75007 Sweden; 2 Faculty of Agricultural Sciences, Agricultural University of Iceland, Hvanneyri, Borgarbyggð, 311 Iceland; 3 Department of Veterinary Science, University of Parma, Parma, 43126 Italy; 4 Center for Animal Breeding and Genetics, Department of Biosystems, KU Leuven, Leuven, 3001 Belgium

**Keywords:** Runs of homozygosity, Heterozygosity, Effective population size, Genomic inbreeding, Performance, Adaptation, Metabolism, Immune system, Coat colours

## Abstract

**Background:**

The Icelandic horse and Exmoor pony are ancient, native breeds, adapted to harsh environmental conditions and they have both undergone severe historic bottlenecks. However, in modern days, the selection pressures on these breeds differ substantially. The aim of this study was to assess genetic diversity in both breeds through expected (H_E_) and observed heterozygosity (H_O_) and effective population size (Ne). Furthermore, we aimed to identify runs of homozygosity (ROH) to estimate and compare genomic inbreeding and signatures of selection in the breeds.

**Results:**

H_O_ was estimated at 0.34 and 0.33 in the Icelandic horse and Exmoor pony, respectively, aligning closely with H_E_ of 0.34 for both breeds. Based on genomic data, the Ne for the last generation was calculated to be 125 individuals for Icelandic horses and 42 for Exmoor ponies. Genomic inbreeding coefficient (F_ROH_) ranged from 0.08 to 0.20 for the Icelandic horse and 0.12 to 0.27 for the Exmoor pony, with the majority of inbreeding attributed to short ROHs in both breeds. Several ROH islands associated with performance were identified in the Icelandic horse, featuring target genes such as *DMRT3*, *DOCK8*, *EDNRB*, *SLAIN1*, and *NEURL1*. Shared ROH islands between both breeds were linked to metabolic processes (*FOXO1*), body size, and the immune system (*CYRIB*), while private ROH islands in Exmoor ponies were associated with coat colours (*ASIP*, *TBX3*, *OCA2*), immune system (*LYG1*, *LYG2*), and fertility (*TEX14*, *SPO11*, *ADAM20*).

**Conclusions:**

Evaluations of genetic diversity and inbreeding reveal insights into the evolutionary trajectories of both breeds, highlighting the consequences of population bottlenecks. While the genetic diversity in the Icelandic horse is acceptable, a critically low genetic diversity was estimated for the Exmoor pony, which requires further validation. Identified signatures of selection highlight the differences in the use of the two breeds as well as their adaptive trait similarities. The results provide insight into genomic regions under selection pressure in a gaited performance horse breed and various adaptive traits in small-sized native horse breeds. This understanding contributes to preserving genetic diversity and population health in these equine populations.

**Supplementary Information:**

The online version contains supplementary material available at 10.1186/s12864-024-10682-8.

## Background

Monitoring the genetic diversity within populations is vital to ensure sustainable breeding and should be performed routinely within breeding programs [1, 2]. This especially applies to breeding programs involving closed populations, such as the Icelandic horse and the Exmoor pony breeds. Both breeds are ancient, native breeds adapted to harsh environmental conditions, and they have both undergone severe historic bottlenecks, albeit more pronounced in the Exmoor pony breed. However, in modern days, the selection pressures on these breeds differ substantially. The Icelandic horse has been bred primarily for its performance in five gaits, while conservation efforts have been the focus for the endangered Exmoor pony. Despite their similar starting points, the divergent breeding goals offer a unique opportunity to study the effects of artificial and natural selection by comparing the genomic selection signatures in these two breeds.

Little is known with certainty about the origin of the Icelandic horse breed, but it is generally believed to have descended from horses brought to the country by Norse settlers around 1100 years ago [3]. Since the settlement, the horses have remained isolated in Iceland and survived harsh weather conditions and natural disasters, such as volcanic eruptions, without significant introduction of foreign genetic material [3, 4]. Before selective breeding started in the 20th century, the horse was mainly used for labour and transportation and was primarily shaped by its harsh natural habitat. In the 1950s, the first official breeding goal, emphasising a versatile riding horse with five gaits, was introduced, resulting in a shift in the selection criteria for the breed [4]. Selective breeding became prevalent, and already in the 1980s, the official breeding program adopted the method of best linear unbiased prediction (BLUP) animal model to estimate breeding values [5, 6]. In the wake of selective breeding and the increased global popularity of the breed during the late 20th century, particularly in northern Europe, the population size surged. In 1959, the population counted approximately 30,000 horses [7], but to date, approximately 300,000 horses are registered across 31 countries [8].

The Exmoor pony, much like the Icelandic horse, is an ancient native breed adapted to harsh conditions. A stud book for the Exmoor pony was established in 1921 to promote the breeding of purebred Exmoor ponies and ensure they retain the traits and characteristics of their ancestors [9]. However, the breed faced a severe population bottleneck during World War II, dwindling to about 50 individuals. Consequently, conservation efforts have prioritized the Exmoor pony, implementing a breeding program specifically designed for its preservation [9]. The breed is named after the high moorland in north-western Somerset and northern Devon, England, where these ponies traditionally roam free. However, Exmoor ponies are also bred at other sites in the UK, Europe, and North America. Today, there are approximately 500 ponies on Exmoor and an additional 3500 Exmoor ponies in various locations across the UK and other countries [9]. About 500 breeding mares and 100 licensed, registered stallions globally produce between 100 and 150 foals annually. Each foal born to registered parents is inspected by trained inspectors to ensure that the Exmoor pony’s characteristics and traits are maintained. The Exmoor pony breeding, therefore, focuses on maintaining breed standards, particularly regarding exterior features like coat colour and conformation [9].

Natural and artificial selection tends to reduce genetic variability within targeted genomic regions, resulting in increased homozygosity. These so-called signatures of selection in the genome can be studied using modern genomic methods, such as estimations of continuous homozygous segments called runs of homozygosity (ROH) [10, 11]. To date, estimates of ROH have been used to identify genomic regions potentially under artificial selection in multiple horse breeds. Several genomic regions associated with selection for athletic performance have been identified [12–16], and previously documented target gene (*DMRT3*) related to gait pattern has been confirmed [17, 18]. Furthermore, genomic regions associated with selection for complex traits such as temperament, disease susceptibility, and fertility have been suggested [12, 13, 17–21] as well as regions associated with coat pigmentation characteristics and morphological traits such as body size [17, 18, 21–23].

ROH can be caused by the mating of related animals and are, therefore, a measure of inbreeding [10, 11]. In general, short ROHs indicate distant inbreeding, but longer ROHs (> 5.0 Mb) suggest more recent inbreeding where the common ancestor occurs approximately up to 10 generations back [10]. The genomic inbreeding coefficient F_ROH_ is defined as the proportion of the autosomal genome that lies within ROH above a specified length [24]. Recent studies on different horse breeds have reported F_ROH_ estimates to range from 0.10 to 0.29 in breeds with closed stud books [14, 18–23, 25, 26]. In contrast, much lower coefficients have been estimated in breeds with semi-open stud books, such as the Swedish Warmblood horse (F_ROH_ = 0.006) [12].

A recent estimate of the mean pedigree-based inbreeding coefficient (F_PED_) for all Icelandic horses born in Iceland 2020, was reported to be 0.03 [27]. The effective population size (Ne) for the same cohort was estimated to range from 95 to 103 horses depending on the pedigree completeness index [27]. Inbreeding coefficients for the Icelandic horse population have also been estimated using genomic data. An estimate of the average genomic inbreeding coefficient based on microsatellite data was 0.04 [7], while those based on medium-density single-nucleotide polymorphism (SNP) data ranged from 0.08 to 0.13 [16, 18, 28–30]. For the Exmoor ponies, estimated genomic inbreeding coefficients have been reported to range from 0.17 to 0.25 [12, 18, 29].

Genomic data has furthermore been used to estimate the effective population size of both the Icelandic horse and Exmoor pony breeds. For the Icelandic horse, the Ne estimates varied depending on the type of genomic data used: ranging from 215 individuals based on microsatellite data from 442 horses [7] to 555 individuals based on SNP array data from 25 horses [29]. For the Exmoor pony, the Ne was estimated at 216 individuals based on a sample of 24 ponies with SNP array data [29]. Additionally, studies using medium-density SNP array data identified signatures of selection on equine chromosomes (ECA) 3, 10, 11, 15, and 23 in the Icelandic horse [18, 31, 32]. In contrast, a larger number of ROH islands were identified on ECA1-4, 6, 9, 11, 16, 18–19, 22–23, 28, and 30 in Exmoor ponies [17, 18, 31].

Due to inconsistencies in estimates between previous studies regarding especially genetic diversity in the Icelandic horse, as well as indications from pedigree analysis of a decreasing effective population size, updated estimations for this breed based on a larger data set and high-density SNP information are desired. The comparison with the Exmoor pony gives a valuable opportunity to distinguish between detected signatures of selection for performance, and signatures resulting from adaptations to harsh environment.

The aim of this study was therefore to assess genetic diversity and identify runs of homozygosity in the two breeds, and to estimate and compare genomic inbreeding and signatures of selection. We hypothesized that these breeds would share some signatures of natural selection for adaptation in their genomes, whereas signatures of artificial selection for performance would be specific for the Icelandic horse.

## Methods

### Sample collection

The study included 380 privately owned Icelandic horses born between 1993 and 2016, of which 166 were stallions or geldings and 214 were mares. Hair samples were collected from the horses’ tails, and the collection was performed at breeding field tests and visits to trainers and breeders in Iceland and Sweden. The majority of horses were born in Iceland (*N* = 299) and Sweden (*N* = 72), while a few were born in Denmark, Germany, and Norway (*N* = 9). According to previous studies Icelandic horses are well genetically connected within Iceland [7] and across country borders in continental Europe [33], indicating a comparable genetic background of horses in the sample. The sampled individuals were originally selected for different genome-wide association studies; half of the individuals were selected based on mane growth characteristics [34], while the other half was randomly chosen at breeding field tests [35]. All but ten geldings had been shown at a breeding field test and are therefore a part of preselected Icelandic horses more likely to contribute genetic material to future generations [36]. Based on pedigree data from the international Worldfengur database [8], the closest relatedness observed between individuals in the sample were two parent-offspring pairs. In addition, less than 1% of all possible relationships in the dataset were closer than half sibs but less related than full sibs. Efforts were made to balance the contributions from different families and avoid stratification in the data.

Genotype data for 280 Exmoor ponies was retrieved from a previous publication where details concerning data collection are described [37]. The Exmoor ponies were originally selected based on their insect bite hypersensitivity status, avoiding close relatedness as far as possible based on a complete pedigree data four generations back. Furthermore, three subpopulations were reported within the sample set [37].

### Genotype data

The procedure of DNA extraction from the Icelandic horse samples was described in the aforementioned genome-wide association studies [34, 35]. The 380 DNA samples were genotyped with the 670 K + Axiom Equine Genotyping Array. Quality control (QC) was performed using PLINK v1.9 software [38, 39]. For the ROH analysis, poorly genotyped data was removed based on criteria of missing genotypes per SNP (> 0.10) and missing SNPs per sample (> 0.10). No pruning for low minor allele frequency (MAF), deviation from Hardy-Weinberg equilibrium (HWE) or strong LD was done for the ROH analysis as recommended by Meyermans et al. [30]. Criteria for MAF (< 0.05) was however added when calculating the effective population size and observed and expected heterozygosity to be able to compare with similar studies. Only autosomal SNP markers were used for downstream analysis. The genotype data for the 280 Exmoor ponies was also derived from a 670 K Axiom Equine Genotyping Array. The same quality control criteria were used for the genotype data for the Exmoor ponies as for the Icelandic horse data described above. SNP positions were according to genomic coordinates in EquCab3.0 reference genome in both data sets.

After QC including pruning for MAF, the number of SNPs to be used for heterozygosity and Ne analyses was 360,755 and 322,209 for the Icelandic horses and Exmoor ponies, respectively. All the samples for the Icelandic horses passed QC, but six samples from the Exmoor pony group were discarded due to missing genotype data, leaving data for 274 Exmoor ponies for further analyses.

For the ROH analysis, QC was conducted without MAF pruning, resulting in 550,405 shared SNPs for downstream analysis using a combined dataset with information from both breeds.

Principal component analysis (PCA) was performed using the SNPRelate package [40] in R (version 4.3.1) [41] as a QC measure to identify outliers or sample mix-ups in the data as well as to visualize the genetic relationships and clustering patterns in the two breeds. The PCA plot, highlighting the distinct genetic signatures of the two breeds, is presented in Additional file 1: Fig. S1.

### Pedigree analysis

Pedigree data for the Icelandic horse was obtained from the international Worldfengur database [8]. The pedigree data contained information about individuals born from 1860 to 2023, but the earliest records only included a small proportion of the population at that time. The quality of the pedigree data was estimated by calculating the pedigree completeness using the optiSel package [42] in R (version 4.3.1) [41]. The optiSel package was also used to estimate F_PED_ and Ne based on the pedigree data for the 380 Icelandic horses in this study. This package estimates Ne from the mean rate of increase in coancestry. Velie et al. [37] reported that the pedigree data for the Exmoor ponies was complete for four generations. This pedigree data, of lower depth than that for the Icelandic horses, was not available for pedigree analysis in the present study and therefore we focused on genomic analysis for the Exmoor ponies.

### Heterozygosity and effective population size trajectory

Trends in recent Ne trajectories were determined for both breeds using the SNeP v1.1 software [43]. Only Icelandic horses born between 2006 and 2016 (342 horses), and Exmoor ponies born between 1999 and 2009 (148 ponies) were used for the Ne analysis, covering approximately one generation interval. The minimum and maximum distance between pairs of SNPs was set to 0.05 Mb and 40 Mb, respectively, and the alpha value for the formula by Corbin et al. [44] used by the software to estimate Ne from LD was set to 2.2. The recombination rate was furthermore set to 1.24 × 10^− 8^, and the Sved & Feldman approximation [45] was used as a recombination rate modifier. The default value of 0.05 was used as minimum MAF.

Observed (H_O_) and expected heterozygosity (H_E_) was estimated for all the Icelandic horses and all the Exmoor ponies using the --het command in PLINK v1.9 [38, 39].

### Runs of homozygosity and genomic inbreeding

The detectRUNS package [46] in R (version 4.3.1) [41] was used for analysing ROH with a sliding windows approach. The scanning window size was set equal to 10 SNP loci, and the maximum number of heterozygous or missing SNP in the sliding window was set equal to 0. The ROH parameter settings were optimised following recommendations in Meyermans et al. [30]. The final definition of the settings was as follows: (i) maximum distance between consecutive SNPs equal to 100 kb, (ii) minimum SNP density equal to 0.05 SNP/kb, (iii) minimum number of SNP in a run equal to 10 and (iv) minimum length of a run equal to 100 kb. One missing and one heterozygous SNP was allowed per run. The settings allowed ROH detection for 99.4% of the autosomal genome, indicating high validity of the analysis [30]. The minimum length of a run did not affect the genome coverage. Therefore, it was chosen based on the correlations between the F_ROH_ and F_PED_ values for the Icelandic horses, which was highest (*r* = 0.57, *p* < 2.2 × 10^− 16^) when the minimum ROH length was set equal to 100 kb. The identified ROH were divided into five length classes (0.1 < ROH ≤ 1 Mb; 1 < ROH ≤ 2 Mb; 2 < ROH ≤ 4 Mb; 4 < ROH ≤ 8 Mb; and ROH > 8 Mb).

The F_ROH_ was calculated by summing each individual’s total length of ROH and dividing it by the autosomal genome length [24], which was set equal to 2281 Mb, based on the genome length covered by SNPs. F_ROH_ was calculated for each chromosome, length class, and as an average coefficient across the genome for each breed. Furthermore, to facilitate comparison with results from other similar studies, we also calculated the F_ROH_ values for both breeds when the minimum length of a run was set equal to 500 kb instead of 100 kb.

### Signatures of selection and gene ontology

ROH islands that were shared by over 70% of the horses in each breed were determined as signatures of selection for that breed. A threshold of 70%, which is conservative compared to values found in the literature [12, 17, 18, 21–23, 25, 26, 47–49], was used to avoid false positive signatures of selection caused by population history events, such as genetic bottlenecks. The EquCab3.0 genomic coordinates of these regions were used to retrieve candidate gene lists from the genome browser Ensembl (release 110, July 2023) [50]. The candidate gene lists were subjected to a gene ontology (GO) analysis using PANTHER v18.0 (released Aug 2023) [51] to determine significantly enriched biological processes and molecular functions positively selected for in the breeds. Further functional annotation of possible candidate genes was performed using the GeneCards database (version 5.18, Oct 2023) [52, 53]. In addition, the Horse QTLdb (release 51, Aug 2023) [54 was used to identify any overlap with previously identified quantitative trait loci (QTL) in horses.

## Results

### Pedigree analysis

**Fig. 1:**
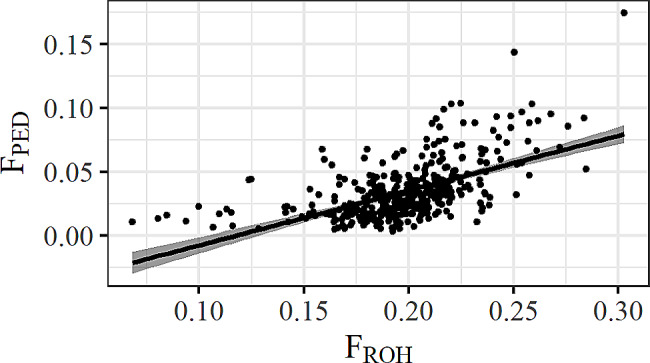
Correlation between F_ROH_and F_PED_. Pearson correlation between F_ROH_ and F_PED_ in the Icelandic horse, with a 95% confidence interval (grey area)

The evaluation of pedigree quality in the Icelandic horse dataset was based on the average number of discrete generation equivalents, resulting in a value of 8.21 (range: 6.15 to 10.1). This value signifies good pedigree completeness. The average F_PED_ for the 380 Icelandic horses in this study was estimated to be 0.03. The comparison between F_ROH_ and F_PED_ revealed a linear relationship (*r* = 0.57, *p* < 2.2 × 10^− 16^) (Fig. 1).

### Heterozygosity and effective population size trajectory

The overall mean H_O_ and H_E_ in the Icelandic horse were equally estimated to be 0.34. In the studied data, H_O_ values ranged from 0.30 to 0.38. Similarly, the H_E_ estimated for the Exmoor pony was 0.34 but the mean H_O_ was 0.33 and ranged between 0.19 and 0.41.

**Fig. 2:**
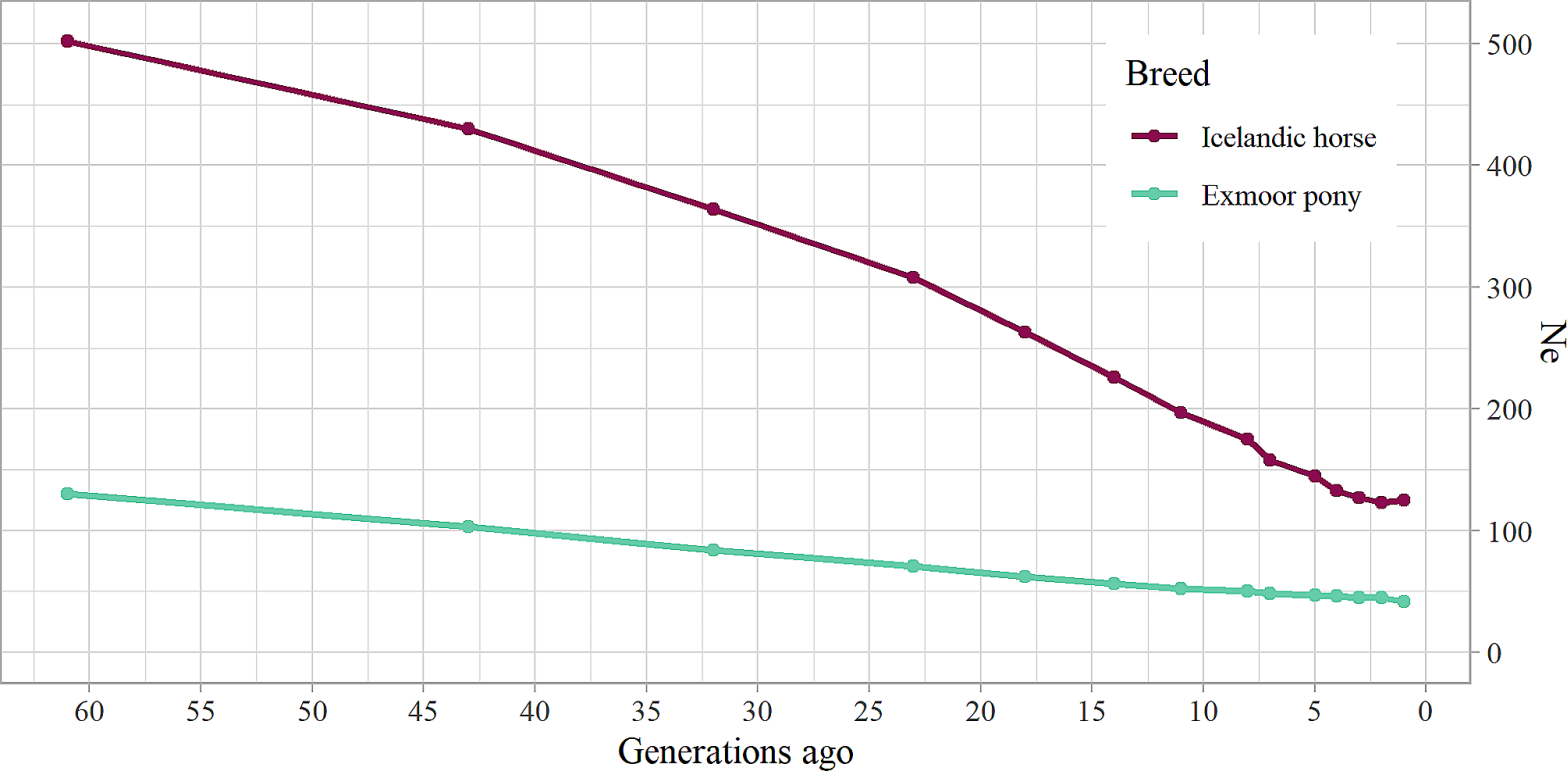
Trends in effective population size Changes in effective population size of the Icelandic horse breed and the Exmoor pony over the last 60 generations based on genomic information

Based on genomic information, the Ne for the last generation of Icelandic horses was estimated to be approximately 125 individuals (Fig. 2). The trend exhibited an overall decline for the past 60 generations, with more pronounced decreases observed around 18–23 and 7–8 generations ago. However, in the most recent 3–4 generations, the Ne trend levelled off, fluctuating within the range of 123 to 127 individuals. In contrast, the genomic Ne estimate for the Exmoor pony was 42 individuals in the last generation. Furthermore, the trend observed for the Exmoor pony over the last 60 generations indicates a consistent, albeit gradual, decrease in Ne.

### Runs of homozygosity and genomic inbreeding

A total of 573,746 and 548,302 ROH were identified for the Icelandic horse and the Exmoor pony, respectively (Table 1). In both cases, the majority of the identified ROH (≥ 96%) was categorised in the shortest length class (0.1 to ≤ 1 Mb) with an average ROH length of 0.24 Mb in the Icelandic horse and 0.26 Mb in the Exmoor pony. The average occurrence of the short ROH was 1455 per individual in the Icelandic horse data and 1921 per individual in the Exmoor pony data. Only 125 Icelandic horses, out of the 380, carried ROH islands categorised in the longest length class (> 8.0 Mb), and on average they carried 2 such ROH islands with a mean length of 10.8 Mb. On the other hand, only one ROH in a single Exmoor pony was identified to belong to the longest length class.

**Table 1:** Descriptive variables from the ROH analysis of the Icelandic horse genome and the Exmoor pony genome

ROH length (Mb)	Icelandic horse						Exmoor pony				
	*N*_ind_	*N*_ROH_	ROH %	S_ROH_	L_ROH_		*N*_ind_	*N*_ROH_	ROH %	S_ROH_	L_ROH_
0.1 to ≤ 1	380	552,929	96.4%	1455	0.24		274	526,242	96.0%	1921	0.26
> 1 to ≤ 2	369	15,677	2.7%	43	1.34		255	19,052	3.5%	75	1.33
> 2 to ≤ 4	335	3,781	0.7%	11	2.66		201	2,879	0.5%	14	2.53
> 4 to ≤ 8	197	1,084	0.2%	5.5	5.40		56	128	0.0%	2.3	4.81
> 8	125	275	0.0%	2.2	10.8		1	1	0.0%	1.0	9.21

N_ind_ = Number of animals, N_ROH_ = total number of ROH, ROH % = relative percentage, S_ROH_ = average number of ROH per animal, L_ROH_ = average length of total number of ROH

**Fig. 3:**
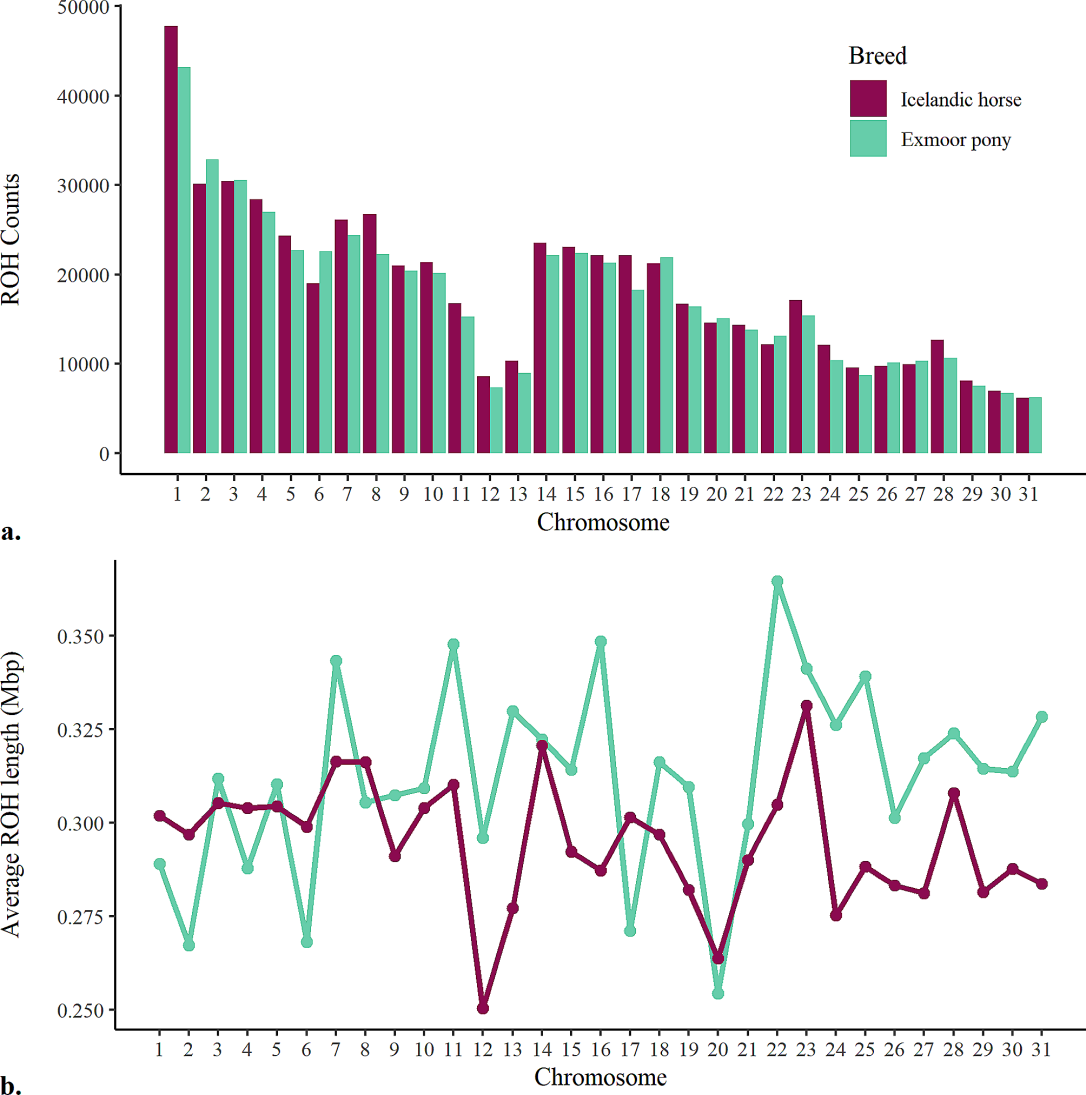
Distribution and average length of ROH across chromosomes (**a**) Distribution and (**b**) average length of ROH in Mb detected across the autosomal genome in the Icelandic horse and the Exmoor pony

ROH quantity, distribution, and average length were estimated per chromosome in both breeds (Fig. 3). The analysis revealed the highest number of ROH in both breeds on ECA1 (N_ROH_ = 47,793 in the Icelandic horse, N_ROH_ = 43,203 in the Exmoor pony), and the lowest on ECA31 (N_ROH_ = 6212 in the Icelandic horse, N_ROH_ = 6227 in the Exmoor pony). For Icelandic horses, ECA23 had the longest average ROH (L_ROH_ = 0.33 Mb), while the shortest (L_ROH_ = 0.25 Mb) were found on ECA12. In Exmoor ponies, the longest average ROH was on ECA22 (L_ROH_ = 0.36 Mb), and the shortest on ECA20 (L_ROH_ = 0.25 Mb).

**Fig. 4:**
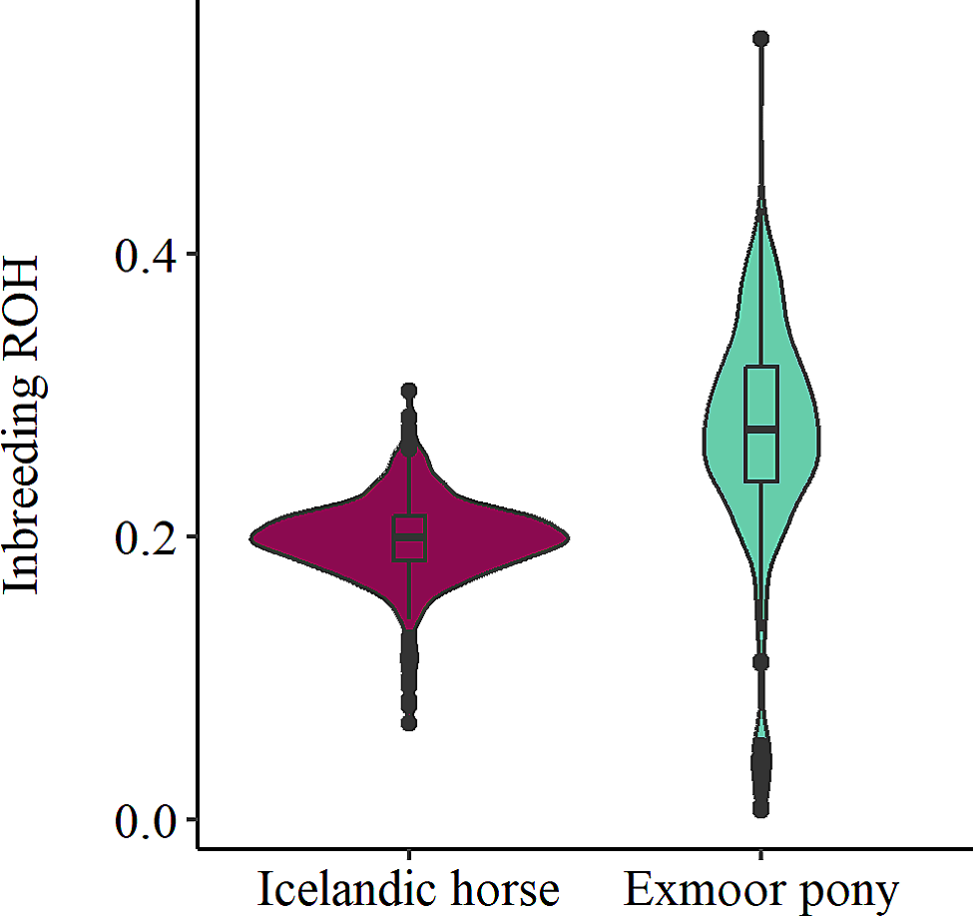
Violin plot showing distribution of genome-wide F_ROH_ Distribution of average F_ROH_ across the genome for the Icelandic horse (to the left) and the Exmoor pony (to the right) represented with a violin plot including a box plot indicating the median, first and third quartile (Q1 and Q3) and the outliers

The estimated mean F_ROH_ was relatively high in both breeds, with a total of 0.20 in the Icelandic horse and 0.27 for the Exmoor pony (Table 2) when including ROH lengths from 100 kb and higher. The individual F_ROH_ ranged from 0.07 to 0.30 for the Icelandic horses, and from 0.01 to 0.55 for the Exmoor ponies. The distribution of average F_ROH_ values across the genome in both breeds is shown in a violin plot in Fig. 4. F_ROH_ estimations for the different ROH length classes revealed that most of the inbreeding could be traced back to the high amount of ROH identified in the shortest length class (0.1 to ≤ 1 Mb) in both breeds. The length classes comprising longer ROH (> 4 to ≤ 8 Mb, and > 8 Mb) accounted for low amount of inbreeding in the Icelandic horse, with F_ROH_ values of 0.01 for each of these two ROH length classes, respectively. In the Exmoor pony, close to zero inbreeding was estimated based only on longer ROH (> 4 Mb).

**Table 2:** Results for each breed’s mean F_ROH_ across the genome and the mean F_ROH_ across the five length classes

ROH length (Mb)	Icelandic horse F_ROH_					Exmoor pony F_ROH_			
	Mean	Min	Max	sd		Mean	Min	Max	sd
0.1 to ≤ 1	0.15	0.07	0.21	0.02		0.22	0.01	0.41	0.06
> 1 to ≤ 2	0.02	0.00	0.06	0.01		0.04	0.00	0.12	0.03
> 2 to ≤ 4	0.01	0.00	0.05	0.01		0.02	0.00	0.08	0.01
> 4 to ≤ 8	0.01	0.00	0.06	0.01		0.00	0.00	0.01	0.00
> 8	0.01	0.00	0.05	0.01		0.00	0.00	0.00	na
**All ROH lengths**	**0.20**	**0.07**	**0.30**	**0.03**		**0.27**	**0.01**	**0.55**	**0.08**

Mean = average F_ROH_ value, Min = minimum F_ROH_ value, Max = maximum F_ROH_ value, sd = standard deviation

The analysis across chromosomes for the Icelandic horse revealed the highest mean F_ROH_, including all ROHs (≥ 0.1 Mb), on ECA23 (F_ROH_ = 0.27 ± 0.09, max = 0.74, min = 0.10) and the lowest mean F_ROH_ on ECA12 (F_ROH_ = 0.15 ± 0.07, max = 0.56, min = 0.02) and ECA20 (F_ROH_ = 0.15 ± 0.07, max = 0.58, min = 0.03). For the Exmoor pony, the highest mean F_ROH_ was identified on ECA22 (F_ROH_ = 0.34 ± 0.17, max = 0.97, min = 0.01) and ECA23 (F_ROH_ = 0.35 ± 0.17, max = 0.92, min = 0.00) and the lowest mean F_ROH_ on ECA12 (F_ROH_ = 0.22 ± 0.13, max = 0.67, min = 0.00) and ECA20 (F_ROH_ = 0.21 ± 0.13, max = 0.78, min = 0.00). A violin plot of mean genomic inbreeding across chromosomes within each breed is shown in Additional file 2: Fig. S2.

When the minimum length of ROH was set equal to 500 kb instead of 100 kb, and thus not including the shortest ROH (0.1–0.5 Mb), the average F_ROH_ for the Icelandic horse was 0.08 and it was 0.12 for the Exmoor pony. Details of F_ROH_ estimates within different length classes from this analysis are shown in Additional file 3: Table S1.

### Signatures of selection and gene ontology

**Fig. 5:**
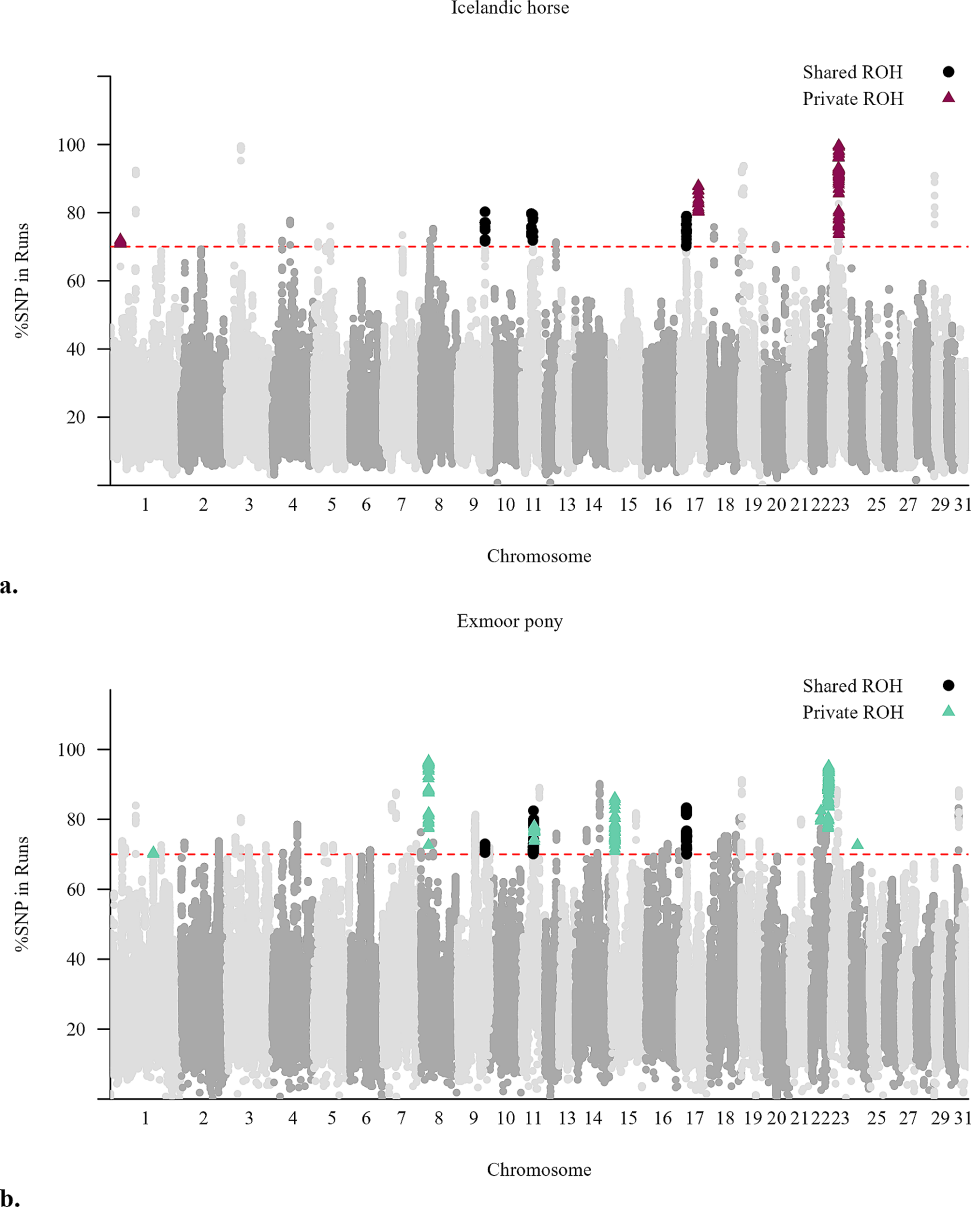
Manhattan plot showing ROH islands across chromosomes ROH islands across all autosomes in (**a**) the Icelandic horse and (**b**) the Exmoor pony. The x-axis represents the chromosome numbers, and the y-axis represents the proportion of animals sharing a ROH. The ROH islands exceeding the 70% threshold (red dotted line) were considered signatures of selection

A total of 15 chromosomes (ECA1, ECA3-5, ECA7-9, ECA11-12, ECA17-20, ECA23, and ECA29) contained ROH islands that were shared by more than 70% of the individuals in the Icelandic horse sample (Fig. 5a), while a total of 23 chromosomes (ECA1-9, ECA11-12, ECA14-19, ECA21-24, and ECA30-31) contained ROH islands shared by more than 70% of the Exmoor ponies (Fig. 5b). The most prominent ROH island hot spot in the Icelandic horse, shared by over 90% of the individuals was located on ECA23 in the region where the *DMRT3* gene is located. On the other hand, the most prominent ROH island hot spot in the Exmoor pony, also shared by over 90% of the sampled individuals, was on ECA22. A complete list of all identified ROH islands for both breeds is shown in Additional file 4: Table S2. The two breeds had overlapping ROH islands on six chromosomes (ECA1, ECA3, ECA8, ECA9, ECA17 and ECA19). The list of annotated genes within and in the vicinity of these shared ROH islands is shown in Additional file 5: Table S3.

Overall, 37 annotated genes were located within the identified ROH islands in the Icelandic horse and 289 more in their ± 500 kb vicinity. In the Exmoor pony, 181 annotated genes were identified within the ROH islands, and 645 more in their ± 500 kb vicinity. Given the considerable number of ROH islands detected, we decided to concentrate on specific regions that could be associated with traits in either or both of the studied breeds in this article.

Table 3 presents the private ROH islands identified in the Icelandic horse that were linked to performance traits, and genes within those ROH. Similarly, Table 4 outlines the private ROH islands identified in the Exmoor ponies and associated genes that are related to coat colours, fertility, hypertension, and the immune system. Additionally, Table 5 enumerates the shared ROH islands between the two breeds suggested to be associated with the immune system, metabolism, coat type, and body size.

**Table 3:** List of selected private ROH islands in the Icelandic horses with annotated genes located within each ROH island and traits possibly associated with the genes or regions

ECA	Start to end position^1^	Length (kb)	nSNP	Annotated genes within ROH island	Suggested associated trait(s)^2^
1	26,821,929 − 26,922,176	100.2	19	*SH3PXD2A*,* NEURL1*	Learning and memory
17	50,290,519 − 50,523,326	232.8	36	*SLAIN1*,* EDNRB*	Performance
23	21,584,553 − 21,696,531	112.0	21	*PGM5*,* ENSECAG00000003227*	Gaits, performance
	21,771,215 − 21,877,092	105.9	17	*DOCK8*	
	22,117,843 − 22,706,518	588.7	117	*KANK1*,* DMRT1*,* DMRT3*,* DMRT2*	

ECA = equine chromosome, Length (kb) = length of a ROH in kilobase, nSNP = number of SNP in a ROH

^1^Positions are according to genomic coordinates in EquCab3.0 reference genome

^2^Based on HorseQTL database and functional annotations

**Table 4:** List of selected private ROH islands in the Exmoor pony with annotated genes located within each ROH island and traits possibly associated with the genes or regions

ECA	Start to end position^1^	Length (kb)	nSNP	Annotated genes within ROH island	Suggested associated trait(s)^2^
1	114,371,997 − 114,398,670	26.7	5	*OCA2*	Coat colour
	114,458,823 − 114,477,224	18.4	5		
8	20,588,303 − 20,896,604	308.3	50	*TBX3*,* TBX5*	Coat colour
11	33,050,441 − 33,198,699	148.3	22	*TEX14*	Fertility
15	10,154,743 − 10,399,931	245.2	38	*REV1*,* EIF5B*,* TXNDC9*,* LYG1*,* LYG2*	Immune system
22	25,912,035 − 26,060,652	148.6	12	*ENSECAG00000055038*,* ASIP*	Coat colour
22	44,748,478 − 44,900,296	151.8	41	*BMP7*,* ENSECAG00000038425*,* SPO11*	Hypertension and fertility
22	46,065,950 − 46,306,096	240.1	50	*NPEPL1*,* ENSECAG00000004696*,* ENSECAG00000040282*,* GNAS*	Hypertension
	46,313,381 − 46,480,417	167.0	41	*NELFCD*,* CTSZ*,* TUBB1*,* ENSECAG00000060119*,* PRELID3B*	
24	16,374,545 − 16,425,073	50.5	4	*ENSECAG00000031483*,* ENSECAG00000036606*,* MED6*,* ENSECAG00000041394*	Fertility

ECA = equine chromosome, Length (kb) = length of a ROH in kilobase, nSNP = number of SNP in a ROH.

^1^Positions are according to genomic coordinates in EquCab3.0 reference genome.

^2^Based on HorseQTL database and functional annotations.

**Table 5:** List of selected shared ROH islands in the Icelandic horses (ICE) and the Exmoor pony (EXM) with annotated genes located within each ROH island and traits possibly associated with the genes or regions

ECA	Breed	Start to end position^1^	Length (kb)	nSNP	Annotated genes within ROH island	Suggested associated trait(s)^2^
9	ICE	73,198,547 − 73,286,648	88.1	14	*ENSECAG00000023276*,* CYRIB*	Immune system
	EXM	73,072,557 − 73,156,479	83.9	5	*ENSECAG00000022588*,* ENSECAG00000046146*,* ENSECAG00000053909*	
11	ICE	25,277,282 − 25,336,522	59.2	9	*ABI3*,* ZNF652*	Coat type and body size
		29,120,371 − 29,172,299	51.9	8	*//*	
		29,181,352 − 29,237,132	55.8	5	*//*	
	EXM	30,266,841 − 30,334,358	67.5	12	*MMD*	
		30,371,968 − 30,440,645	68.7	8	*SMIM36*	
		30,471,631 − 30,600,164	128.5	21	*TMEM100*	
		30,722,954 − 30,853,756	130.8	16	*ENSECAG00000048512*	
		30,906,432 − 30,998,404	92.0	13	*ANKFN1*	
		31,320,686 − 31,495,910	175.2	26	*NOG*	
17	ICE	18,706,560 − 18,829,942	123.4	17	*FOXO1*	Metabolism
	EXM	18,735,602 − 18,829,942	94.3	14		

ECA = equine chromosome, Length (kb) = length of a ROH in kilobase, nSNP = number of SNP in a ROH

^1^Positions are according to genomic coordinates in EquCab3.0 reference genome

^2^Based on HorseQTL database and functional annotations

## Discussion

Disentangling genomic adaptation from natural and artificial selection within a genome is a challenging task. Some approaches include population genetic analysis, functional genomics, examination of historical data on breeding practices, and genomic comparisons. The Icelandic horse and Exmoor pony share many similarities, being ancient breeds of relatively small body size adapted to harsh conditions, but they also exhibit significant differences due to stringent selection for gait performance in Icelandic horses and emphasis on coat colour and conservation of Exmoor ponies. The comparison of the two breeds, therefore, gives a valuable opportunity to distinguish between detected signatures of selection for performance, and signatures resulting from adaptations to harsh environment. Furthermore, this study is the first to analyse genetic diversity and ROHs in the Icelandic horse using high-density SNP-marker data.

A substantial number of ROH islands, considered indicative of selection signatures, were identified in both breeds, and presented in Additional file 4: Table S2. However, in this discussion we focus on selected regions linked to specific traits relevant to the studied breeds. Shared ROH islands between both breeds were identified, associated with metabolic processes, body size, and the immune system. Additionally, private ROH islands linked to performance in the Icelandic horse and ROH islands associated with coat colours, hypertension, and fertility in the Exmoor pony were identified.

### Exploring genetic diversity

Our findings revealed similar heterozygosity estimates in both the Icelandic horse (mean H_O_ = 0.34) and the Exmoor pony (mean H_O_ = 0.33), which were also comparable to estimates reported in other breeds with closed populations. In a study by Cosgrove et al. [16], which estimated heterozygosity for various breeds in the development of the 670k genotyping array [55], reported average H_O_ = 0.34 ± 0.02 for pony breeds, H_O_ = 0.33 ± 0.01 for draft horse breeds, and H_O_ = 0.32 ± 0.01 for the Icelandic horse.

Estimations of SNP based Ne of the Icelandic horse (125 individuals) further suggested an adequate genetic diversity within the breed, and a relative stability in Ne estimations over the last 3–4 generations. The equal estimations of H_E_ and H_O_ and the low number of longer ROH support the stability observed in the Ne trend in recent generations, and the absence of strong recent inbreeding. In contrast, the Ne estimate for the Exmoor pony (42 individuals) indicates severe loss of genetic diversity, posing a risk to the breed’s sustainability. This decline is likely linked to the grave bottleneck experienced during the Second World War when the pony population diminished to about 50 individuals by the war’s end [9]. While the bottleneck may not be explicitly evident in the Ne trend depicted in Fig. 2, the gradual, slow decrease suggests that conservation measures applied after the war may have been useful. However, there were a few Exmoor ponies with unexpectedly low F_ROH_ in the present study and the subpopulations that Velie et al. [37] identified in the Exmoor data, the relatively small sample size, and the lack of background information prevented us from drawing any definitive conclusions for the Ne trend.

Seen over a longer time span for the Icelandic horse, Ne estimations revealed a significant decrease at two historical events, resulting in a major decline in the population size of the Icelandic horse [7]. The first event occurred approximately 23 generations ago, aligning with the end of the 18th century considering a generation interval of roughly 10 years, coinciding with the *Skaftáreldar* volcanic eruption. This eruption had detrimental consequences for both humans and livestock in Iceland, and is said to have reduced the number of horses with 75% [56]. The second event took place around 8 generations ago, during the industrial revolution in Iceland. During this period, the role of the Icelandic horse as a working horse was superseded by machines, leading to a shift in the breeding goal towards breeding riding horses [4, 57].

While the Ne estimates for the Icelandic horse exceed the generally recommended minimum size for sustaining genetic diversity in selectively bred populations, previous studies have shown a decline in pedigree-based Ne over generations. For instance, in 1989, the pedigree-based Ne was reported as 365 individuals [58], declining to 210 animals by 2000 [59], and currently estimated at around 100 individuals [27]. These figures differ somewhat with the Ne estimates from genomic data presented in Fig. 2 of this study. The pedigree completeness for the Icelandic horse is high, but pedigree measures are limited to probability estimates based on documented ancestry. In contrast, genomic data provides a more detailed and comprehensive view of the population’s genetic structure and history but is based on a genotyped subset of the population. Therefore, some differences in pedigree-based and genomic estimates can be expected. However, it is reassuring that the differences were not substantial.

The average F_PED_ estimated in the present study closely aligns with recent calculations for all horses born in Iceland between 2011 and 2020 [27]. This suggests that our sample predominantly consisting of preselected Icelandic breeding horses likely to contribute to future generations fairly accurately represents the latest generation of Icelandic horses. A continued monitoring of relatedness and genetic contribution of breeding animals, and resulting inbreeding trends, is important to ensure a sustainable breeding program. This is especially important within the Icelandic horse population, because a large part of the population is geographically isolated in Iceland where importation of genetic material is prohibited according to the Animal Importation Act [60].

### Interpreting genomic inbreeding

The quantity of detected ROH islands in this study was heavily influenced by the parameter settings, notably the minimum ROH length. Many equine studies using 670k SNP data set a minimum ROH length of 500 kb [17, 19, 21, 22, 26], excluding the shortest ROHs (< 0.5 Mb). This exclusion further impacts F_ROH_ estimation, which is derived from the total genome length covered by ROHs. In our study, a 100 kb minimum ROH length resulted in the highest correlation between estimated F_ROH_ and F_PED_ values for the Icelandic horse. Using this setting, the mean F_ROH_ was higher (F_ROH_ = 0.20 for the Icelandic horse and F_ROH_ = 0.27 for the Exmoor pony) compared to previous studies. Previous reports for the Exmoor pony ranged from 0.17 to 0.25 [12, 18], while the disparity in F_ROH_ values for the Icelandic horse was more pronounced, with previous estimates ranging from 0.03 to 0.13 [16, 18, 30]. Excluding the shortest ROHs (< 0.5 Mb), resulted in values closer to those previously reported. It can be argued that the F_ROH_ estimate based on the longer ROHs, reflecting more recent inbreeding, is more relevant for risk assessment of the current breeding practises [61, 62].

The high average F_ROH_ observed in the Icelandic horse when including the shortest ROHs, may be attributed to the breed’s assumedly limited initial genetic pool, potential drift, and genetic purging during its adaptation process. The relatively small number of ROHs longer than 4 Mb suggests no evidence of recent excessive inbreeding. However, when compared with the near absence of long ROHs in the Exmoor pony, there is suggestive evidence of a stronger recent selection in the Icelandic horse. Additionally, there are indications of an increased contribution of a limited number of breeding animals in the Icelandic horse to the modern gene pool [27], emphasizing the importance of closely monitoring inbreeding and genetic diversity in the breed.

### ROH island cold spots of different origin

ROH island cold spots were identified on ECA12 and ECA20 in both breeds, where the shortest average ROHs and the lowest mean F_ROH_ were found. The cold spot on ECA20 may be attributed to the major histocompatibility complex (MHC) covering a substantial portion of the chromosome [63, 64]. The MHC is a highly variable region associated with the immune system and benefits from heterozygosity [65], with the Icelandic horse, for instance, showing high MHC heterozygosity [66]. Furthermore, a possible cause of the cold spot identified on ECA12 is the higher percentage of the chromosome covered by copy number variation (CNV) gains and losses compared to other equine chromosomes [67–69]. CNV increases genetic diversity by varying the number of copies of genomic regions [70], indicating higher heterozygosity in the region on ECA12.

### Performance-linked ROH islands on ECA23 and ECA17 in the Icelandic horse

The most prominent ROH island hot spot in the Icelandic horse was located on ECA23; a region harbouring genes such as the *DMRT3* and *DOCK8*, both known to be causative or highly associated with gaits and performance in many horse breeds [71–78]. A single mutation [DMRT3:Ser301STOP marker at nucleotide position 22,999,655 on ECA23] in the *DMRT3* gene, also referred to as the ‘Gait keeper’ mutation, alters the pattern of locomotion and has a predominant effect on gaiting ability in Icelandic horses [71, 72]. The identified ROH harbouring the *DMRT3* gene was the longest ROH (589 kb) identified in this study that was shared by over 70% of the Icelandic horses, indicating recent selection for this region.

This ROH also harbours the *DMRT1*, *DMRT2* and *KANK1* genes and overlaps the ‘Gait keeper’ haplotype previously identified [71, 79]. Furthermore, this region overlaps a previously identified selection signature for the Icelandic horse in a study by Petersen et al. [31]. The *DOCK8* gene, located in another ROH (106 kb) on ECA23, has been shown to be associated with harness racing success in Nordic trotters [73]. Furthermore, in a small sample set of Icelandic horses, the *DOCK8* gene was found to be associated with pace racing success and to potentially segregate between elite pace racers and other horses [80]. Previous studies hypothesized overlapping or common gene effects of the *DMRT1-3* genes and the *DOCK8* gene [73, 81, 82].

Even though the ‘Gait keeper’ mutation has been shown to be a causative factor for gaiting ability, it is highly unlikely that it is the single cause as shown by multiple studies [35, 71, 72, 74, 76–78, 83]. It is therefore possible that the *DOCK8* gene contributes to the performance of gaits, alongside the *DMRT3* gene. The *PGM5* gene, located in the third ROH (112 kb) on ECA23, has no known association with performance in horses. It is predicted to enable metal ion binding activity and phosphoglucomutase activity, and to be associated with myofibril assembly and striated muscle tissue development in zebrafish [84], and may thus be a candidate to study further for performance in horses.

A relatively long ROH island (233 kb) was detected on ECA17 for the Icelandic horses, harbouring the genes *EDNRB* and *SLAIN1*. The *EDNRB* gene harbours the ‘Overo allele’, which has been shown to be the causative factor for the Overo coat colour in horses and the lethal white foal syndrome (LWFS) in homozygous form [85–87]. Since there are no reports of either the Overo coat colour or the LWFS in the Icelandic horse breed, the apparent selection intensity for this region is likely associated with another function of the gene *EDNRB*, that appears to have pleiotropic effects. The *EDNRB* gene is a part of the endothelin gene family, which plays a crucial role in regulating blood vessel tone and blood pressure [88, 89]. The *EDNRB* interacts with its family members, such as the *EDN3* gene [90–92] suggested to be associated with blood supply regulation in high-performing racing horses [93, 94]. Icelandic horses are trained for high intensity exercises [95, 96], indicating the importance of a robust regulatory system for the distribution of blood to the tissues. This ROH island may, therefore, be a product of selection for performance. The *SLAINI* gene has furthermore been associated with the developing nervous system in mouse embryos [97], indicating a possible importance for performance.

Another possible performance related ROH island was detected on ECA1, where the *NEURL1* and *SH3PXD2A* genes are located. One of the functions of the *NEURL1* gene is hippocampal-dependent synaptic plasticity, which affects learning and memory processes [98, 99]. This region could, therefore, be important for horses trained for performance. These ROH islands on ECA23, ECA17 and ECA1 were not identified in the Exmoor pony genome in this study, further underlining the possible association with performance.

### ROH islands distinguished by coat colour genes in the Exmoor pony

The Exmoor pony is renowned for its distinctive bay coat colour and mealy markings. A ROH island hot spot was identified on ECA22, coinciding with the location of the *ASIP* gene which is responsible for the bay coat colour in horses [100]. Additionally, a prominent signature on ECA8, harbouring the *TBX3* and *TBX5* genes, was observed for this breed. While *TBX3* controls dun coat colour [101], the rarity of dun-coloured Exmoor ponies suggests that the signature likely reflects the high prevalence of the non-dun alleles in the gene.

The *OCA2* gene was identified in a ROH island on ECA1 for Exmoor ponies and is known to be one of the components of the mammalian pigmentary system [102–104]. The gene is a major determinant of brown and/or blue eye colour [103–105] and is hypothesized to be a key control point at which ethnic skin colour variation in humans is determined [106]. Efforts have been made to link this gene to horse colour phenotypes [107, 108] without success so far. The *OCA2* gene consistently emerges as a selection signature in the Exmoor pony genome [17], suggesting its potential association with some of their characteristics, such as the mealy markings. The mealy phenotype has previously been linked to the *EDN3* gene [93], which was also identified in this study, located near another ROH island on ECA22. Consequently, we recommend further exploration of these two candidate genes to ascertain their potential association with the mealy phenotype.

No ROH islands harbouring candidate genes for horse colour phenotypes were identified in the Icelandic horse genome. This absence may be attributed to the breeding goal for the Icelandic horse [109], which has consistently aimed at preserving a diverse range of coat colours, presumably leading to higher variability within the colour loci.

### Signs of adaptation to limited feed supply

One of the most prominent ROH islands shared by the Icelandic horse and the Exmoor pony, harboured the *FOXO1* gene on ECA17. The *FOXO1* gene has been associated with insulin resistance [110–112] which is one of the key components of the equine metabolic syndrome (EMS) [113]. EMS is generally observed in breeds categorized as “easily fed,” which typically require a lower nutritional intake to maintain body weight. These breeds, including the Icelandic horse and Exmoor pony, have historical backgrounds marked by poor feed availability and periods of starvation. The hypothesis suggests that positive selection for this genomic region has historically contributed to the survival of these breeds in harsh winter conditions but may render them less adaptive to lush pastures and high-energy diets, and in some cases, low workload. Low insulin sensitivity, or even insulin resistance, has been reported in both breeds [114, 115].

Another key aspect of EMS involves a susceptibility to laminitis [113], which has been shown to be associated with hypertension in horses [115]. Moreover, hypertension arises from dysfunction in vascular endothelial cells in humans with type 2 diabetes [116], a syndrome considered closely related with EMS [117]. Additionally, the vascular endothelium plays a crucial role in preventing platelet activation and the adhesion of leukocytes to the vascular wall [115]. Within the Exmoor pony genome, a substantial homozygote region on ECA22 was identified, harbouring three distinct ROH islands. In the first ROH, the genes *BMP7* and *SPO11* were identified; the second contained the *NPEPL1* and *GNAS* genes, while the third encompassed the *NELFCD*, *CTSZ*, *TUBB1*, and *PRELID3B* genes. Notably, all genes in the third ROH are associated with the regulation of platelet properties [52, 53, 118, 119]. Moreover, research has linked the *BMP7* gene to diabetes and vascular calcification in humans [120], while the *SPO11* gene has been linked to endothelial dysfunction resulting from exercise-induced DNA damage in horses [94]. Furthermore, mutations in the *GNAS* gene have been established as causative for McCune-Albright syndrome in humans, a condition known to involve endocrinologic anomalies such as Cushing syndrome [121]. Equine Cushing’s disease is recognized in many horse breeds and frequently leads to the development of laminitis [117]. At last, the aforementioned *EDN3* gene, which is a part of the endothelin gene family, is located in a close proximity (> 165 kb) to the ROH islands.

The strong evidence of genes associated both directly and indirectly with vascular endothelin regulation in the specified ECA22 region suggests it could be a signature for positive selection, representing an adaptive trait in Exmoor ponies potentially related to varying feed supply. This study did not identify evidence of positive selection for the same region on ECA22 in the Icelandic horse, however.

### Hot spot on ECA11 potentially linked to harsh climate adaptation

ROH islands were identified on ECA11 in both the Icelandic horse (25,277,282 − 29,237,132) and Exmoor pony (30,266,841 − 31,495,910) within a region that appears to be partly shared among various pony and draft horse breeds [17, 18, 21–23, 31, 47–49]. This region has been shown to have a low recombination rate in horses [122]. It ranges from approximately position 23 Mb to 32 Mb and has predominantly been associated with phenotypes such as a small to medium height at withers, and a compact, muscular body and robust bone structure, as observed in pony and draft horse breeds [123–125], and has also been suggested to be involved in hair and coat density and quality [48, 49, 126].

Whereas phenotypes such as limited height at withers and dense winter coat apply to the Icelandic horse and Exmoor pony, the genes in this wider genomic region previously suggested to be of importance for such traits were not within the ROH islands identified on ECA11 in the present study. However, we cannot exclude that selection has targeted other nearby genes, given the overall high gene density in the region, or that regulatory functions have been selected for. The low recombination rate in the region [122] suggests strong linkage and perhaps participation of many genes in similar processes.

### Adapted antibacterial defence

A ROH island identified on ECA9 (73,072,557 − 73,286,648) shared by both horse breeds may play a crucial role in the immune system. Within this region lies the *CYRIB* gene, which has been shown to be associated with protection against Salmonella bacterial infections in humans and contribute to restricting infections mediated by Mycobacterium tuberculosis and Listeria monocytogenes [127].

Functional annotation analyses of genes found within a ROH island on ECA15 only in the Exmoor pony (10,154,743 − 10,399,931) revealed an enrichment in GO terms related to the “defence response to Gram-positive bacterium”. Gram-positive bacteria include genera like Staphylococcus, Streptococcus, Clostridium, and Listeria, all known to cause diseases of varying severity in horses [128–131]. The genes identified within the ROH include *REV1*, *EIF5B*, *TXNDC9*, *LYG1*, and *LYG2*. The two last ones, *LYG1* and *LYG2*, have been reported to have a significant role in innate immunity in mammals [132]. As far as our knowledge extends, this specific region has not been recognized as a selection signature in other horse breeds, while the *LYG1* and *LYG2* genes have been identified as candidate genes for selection in sheep [133].

### Male fertility related genomic regions in the exmoor pony

Three ROH islands detected in the Exmoor pony genome harbour genes related to male fertility traits. First, a ROH island was detected on ECA11, positioned at 33,050,441 to 33,198,699, containing the *TEX14* gene. Second, a ROH island was identified on ECA22 ranging from 44,748,478 to 44,900,296, harbouring the *SPO11* gene. At last, a ROH detected on ECA24 (16,374,545 − 16,425,073) harboured genes that, by a functional annotation analysis, revealed an enrichment in the GO term related to “male gonad development”.

The *TEX14* gene codes for a testis-specific protein and serves as a crucial element in the intercellular bridges of both male and female embryos. Adult male mice lacking *TEX14* mRNA are unable to reproduce (sterile), while females with the same genetic condition maintain their fertility [134, 135]. *TEX14* has further been suggested to have been targeted by selection for fertility in German warmblood horses [13] and was located within a ROH island detected in the Noriker horse genome [23]. The *SPO11* gene codes for an evolutionarily conserved topoisomerase-like protein that, in mammals, is functionally expressed in gonads during meiosis. It has been shown to be associated with male infertility in mice, humans, and cattle [136–140]. The ROH island on ECA24 contained the *MED6* gene and three novel genes (ENSECAG00000031483, ENSECAG00000036606, ENSECAG00000041394). ENSECAG00000041394 is an orthologue of the *ADAM20* mouse gene. The ADAM metallopeptidase domain 20 (*ADAM20*) gene is specifically expressed in testis and has been associated with male infertility in humans and mice [141–144]. The presence of these ROH islands associated with male fertility implies that this trait may have undergone positive selection in the Exmoor pony breed, as a survival trait in semi-feral conditions. These ROHs were not detected in the Icelandic horse in this study.

## Conclusions

This study provides insights into the genetic diversity and genomic ROH patterns in the Icelandic horse and Exmoor pony. Our assessments indicate that the genetic diversity in the Icelandic horse is on an acceptable level for a closed population undergoing artificial selection. Nevertheless, it is advisable to maintain ongoing monitoring to guarantee the preservation of genetic diversity and to support sustainable breeding practices for the Icelandic horse. In contrast, our results for the Exmoor pony indicates a critical state of genetic diversity. However, further research accounting for the population structure of the breed is needed to validate our findings.

The F_ROH_ estimates were significantly affected by the parameters employed in the ROH analysis, emphasizing the importance of considering these settings when comparing values across different studies. In our study, the high occurrence of short ROHs led us to attribute a larger extent of the identified inbreeding in both breeds to historical events like the breed’s origin, bottlenecks, and adaptation, rather than recent and stringent selection practices.

Several ROH islands associated with performance were identified in the Icelandic horse, effectively distinguishing the breed from the Exmoor pony. The most prominent one on ECA23 featured the longest average ROHs and the highest mean F_ROH_ across all chromosomes, suggesting the most recent and stringent selection pressure. The shared ROH islands observed in both breeds were linked to traits associated with adapting to challenging environments with limited food resources, as well as to immune system function. Conversely, distinct ROH regions specific to Exmoor ponies were associated with their exterior characteristics such as coat colour, along with traits related to immune response and fertility.

In conclusion, this study provides knowledge contributing to preserving genetic diversity and population health in these two equine populations. Furthermore, the obtained results provide important insight into genomic regions shared by the two breeds, which are likely associated with adaptive traits shaped by natural selection. Genomic regions related to performance were identified only in the Icelandic horse, likely reflecting the artificial selection for gaits and performance that has occurred over the past few decades.

## Electronic supplementary material

Below is the link to the electronic supplementary material.

Supplementary Material 1

Supplementary Material 2

Supplementary Material 3

Supplementary Material 4

Supplementary Material 5

## Data Availability

This study did not generate new data; all data used were pre-existing. The Icelandic horse genotypes analysed during the study have been deposited in the European Variation Archive (EVA) [145] at EMBL-EBI under accession number PRJEB74212 (https://www.ebi.ac.uk/eva/?eva-study=PRJEB74212). The Exmoor pony genotypes are available via Figshare (DOI: https://doi.org/10.6084/m9.figshare.3145759).

## Abbreviations

BLUP: Best linear unbiased prediction
CNV: Copy number variation
DNA: Deoxyribonucleic acid
ECA: Equus caballus chromosome
EMS: Equine metabolic syndrome
GO: Gene ontology
HWE: Hardy-Weinberg equilibrium
LD: Linkage disequilibrium
LWFS: Lethal white foal syndrome
MAF: Minor allele frequency
MHC: Major histocompatibility complex
mRNA: Messenger RNA
PCA: Principal component analysis
QC: Quality control
QTL: Quantitative trait loci
RNA: Ribonucleic acid
ROH: Runs of homozygosity
SD: Standard deviation
SNP: Single nucleotide polymorphism
