# Correction: Genetic diversity and signatures of selection in Icelandic horses and Exmoor ponies

**DOI:** 10.1186/s12864-024-10908-9

**Published:** 2024-10-22

**Authors:** Heiðrún Sigurðardóttir, Michela Ablondi, Thorvaldur Kristjansson, Gabriella Lindgren, Susanne Eriksson

**Affiliations:** 1 Department of Animal Biosciences, Swedish University of Agricultural Sciences, P.O. Box 7023, Uppsala, 75007 Sweden; 2 Faculty of Agricultural Sciences, Agricultural University of Iceland, Hvanneyri, Borgarbyggð, 311 Iceland; 3 Department of Veterinary Science, University of Parma, Parma, 43126 Italy; 4 Center for Animal Breeding and Genetics, Department of Biosystems, KU Leuven, Leuven, 3001 Belgium

**Correction:** ***BMC Genomics*** **25, 772 (2024)**

10.1186/s12864-024-10682-8

Following publication of the original article the sentence highlighted in bold was removed from the third paragraph of the Background section:

The Exmoor pony, much like the Icelandic horse, is an ancient native breed adapted to harsh conditions. **This breed has coexisted with humans in Britain for over 30,000 years.** A stud book for the Exmoor pony was established in 1921 to promote the breeding of purebred Exmoor ponies and ensure they retain the traits and characteristics of their ancestors [9].

The original article has been updated.

