# Supplementary material for: Genetic diversity and signatures of selection in Icelandic horses and Exmoor ponies
Source: BMC Genomics. 2024 Aug 8;25:772. doi: 10.1186/s12864-024-10682-8 (PMC11308356; doi:10.1186/s12864-024-10682-8)
Supplement: Supplementary file 1 — Supplementary Material 1 [file 12864_2024_10682_MOESM1_ESM.docx]

**F_ROH_ results with the minimum setting of ROH length as 500 kb.**

| **ROH length**  **(Mb)** | **Icelandic horse F_ROH_** | | | |  | **Exmoor pony F_ROH_** | | | |
| --- | --- | --- | --- | --- | --- | --- | --- | --- | --- |
|  | **Mean** | **Min** | **Max** | **sd** |  | **Mean** | **Min** | **Max** | **sd** |
| **0.5** to ≤ 1 | 0.04 | 0.00 | 0.07 | 0.01 |  | 0.07 | 0.00 | 0.17 | 0.03 |
| > 1 to ≤ 2 | 0.02 | 0.00 | 0.06 | 0.01 |  | 0.04 | 0.00 | 0.12 | 0.03 |
| > 2 to ≤ 4 | 0.01 | 0.00 | 0.05 | 0.01 |  | 0.02 | 0.00 | 0.08 | 0.01 |
| > 4 to ≤ 8 | 0.01 | 0.00 | 0.06 | 0.01 |  | 0.00 | 0.00 | 0.01 | 0.00 |
| > 8 | 0.01 | 0.00 | 0.05 | 0.01 |  | 0.00 | 0.00 | 0.00 | na |
| **All ROH lengths** | **0.08** | **0.00** | **0.21** | **0.04** |  | **0.12** | **0.00** | **0.32** | **0.06** |

Mean = average F_ROH_ value, Min = minimum F_ROH_ value, Max = maximum F_ROH_ value, sd = standard deviation

**Table S1.** Results for each breed's mean F_ROH_ across the genome and the mean F_ROH_ across the five length classes when the minimum length of a run was set equal to 500 kb instead of 100 kp.
