# Supplementary figures and images for: Genetic diversity and signatures of selection in Icelandic horses and Exmoor ponies
Source: BMC Genomics. 2024 Aug 8;25:772. doi: 10.1186/s12864-024-10682-8 (PMC11308356; doi:10.1186/s12864-024-10682-8)

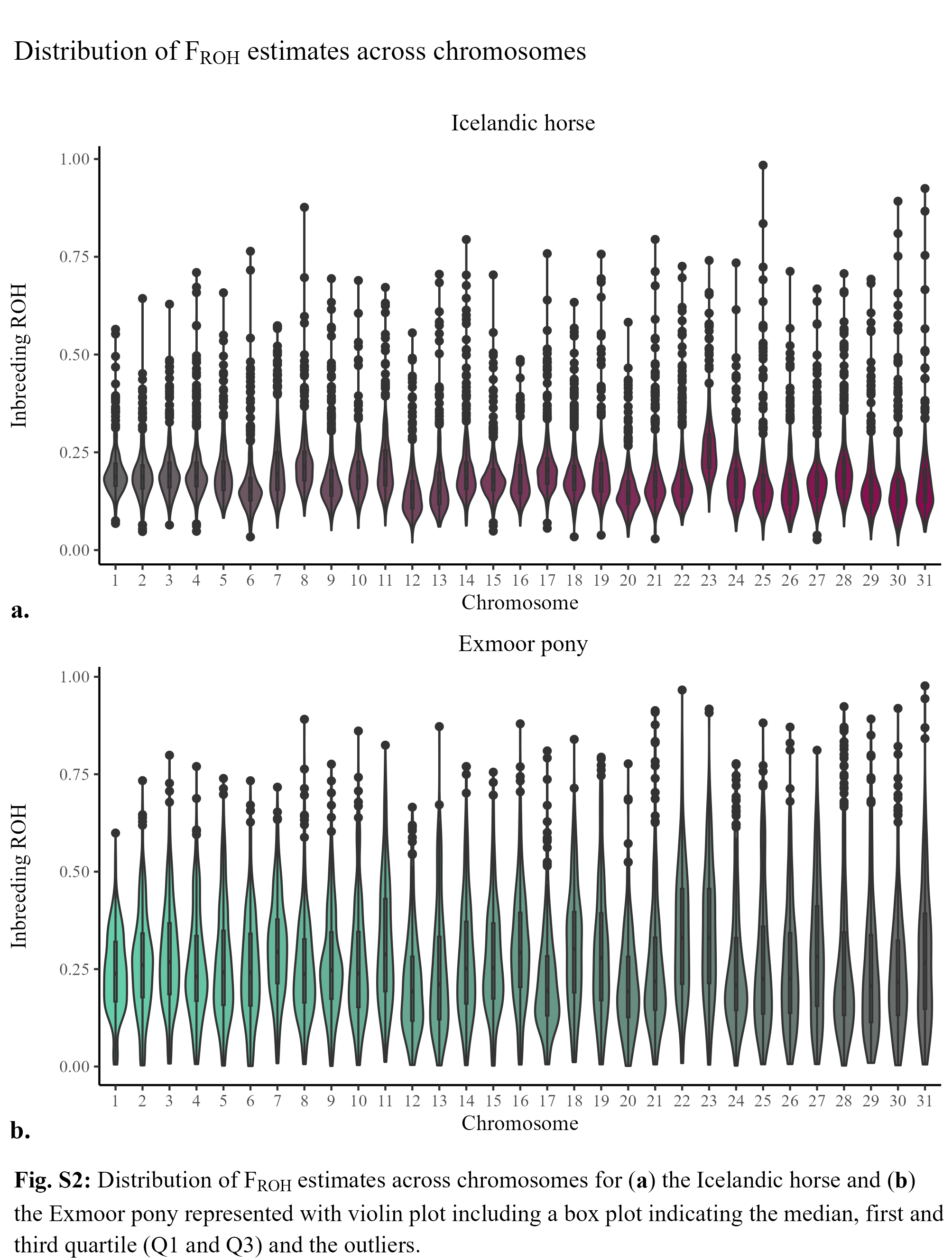

Supplement: Supplementary file 4 — Supplementary Material 4 [file 12864_2024_10682_MOESM4_ESM.png]

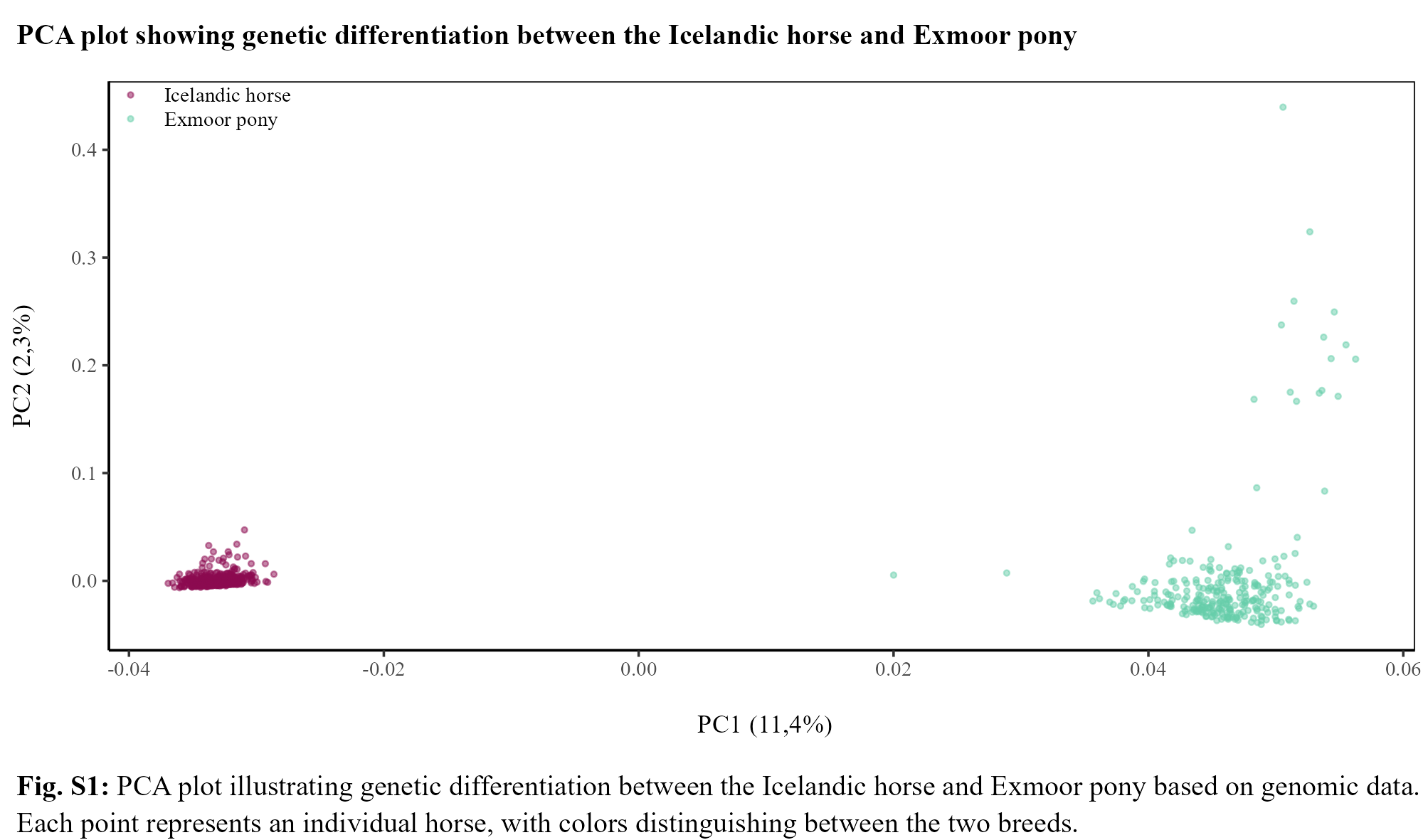

Supplement: Supplementary file 5 — Supplementary Material 5 [file 12864_2024_10682_MOESM5_ESM.png]
